# Entrustability levels of general internal medicine residents

**DOI:** 10.1186/s12909-021-02624-9

**Published:** 2021-03-25

**Authors:** Mostafa Dehghani Poudeh, Aeen Mohammadi, Rita Mojtahedzadeh, Nikoo Yamani

**Affiliations:** 1 Department of Medical Education, School of Medicine, Tehran University of Medical Sciences, Tehran, Iran; 2 Department of E-learning in Medical Education, Virtual School, Center for Excellence in E-learning in Medical Education, Tehran University of Medical Sciences, Tehran, Iran; 3 Department of Medical Education, Isfahan University of Medical Sciences, Isfahan, Iran

**Keywords:** Competence, Competency based medical education, Medical education, Internal medicine, Entrustment, Entrustability, Entrustable professional activities, Assessment, Workplace-based assessment

## Abstract

**Background:**

Entrustable professional activities (EPAs) are those activities that a health professional can perform without direct supervision in a defined environment. Bridging the gap between competencies and learning objectives, EPAs have made assessing the performances of health professional more realistic. The main objective of the present study was developing and customizing EPAs for Iranian Internal Medicine Residency Programs.

**Results:**

After reviewing the publications, residency curricula and logbooks, and collecting experts’ ideas, the initial list of EPAs was developed. Then, in a focus group, the list was refined, the entrustability level of each residency year was determined, and finally, the EPA-competency cross-tab was established, and in the next step, through a one- round Delphi, the results were validated. Twenty-eight EPAs were developed. Some of them were definitely suitable for the higher levels of residency, such that they had to be accomplished under direct supervision until the end of the program. On the other hand, some of EPAs were those that residents, even from the first year, are expected to perform independently or under indirect supervision. Most of the EPAs cover a wide range of competencies.

**Conclusion:**

Determining the entrustability level of each residency year in each EPA as well as the competency- EPA matrix has crucial effect on the quality of the graduates. It seems that our findings are applicable in developing countries like Iran.

## Introduction

Transition to the paradigm of competency-based education, from process- based or time-based one, requires attainment of a shared language and transforming the competencies to such constructs that are not too much subjective [1–3]. It has been unclear what is expected from the learner to perform in the practical context [4]. As a result, the new paradigm has imposed a need to some means and methods that can evidently evaluate the quality of performances in the work places. Such evidences must be compared to the learners’ activities in the clinical settings [5–7]. Moreover, currently, most of the high stake decisions like promoting to a higher level of study and entering the practice are mainly made by means of knowledge based summative assessments, particularly in graduate programs. This is more or less because of the complications regarding the high objective tests [8]. Some of the problems with such examinations like Objective Structured Clinical Exam (OSCE) are the need for advanced plans for time and cost allocation [9]. Therefore, formative work based assessments like mini clinical evaluation examination (mini-CEX) are recently more acknowledged [10, 11]. However, the psychometric investigations of these examinations have revealed different results and conclusions; some with paradoxical results even with questioning the reliability and feasibility of them [12, 13]. Therefore, it is essential to apply instruments capable to measure and evaluate the subjective and complicated constructs of competencies, along with helping medical schools become more socially accountable [14]. Such examinations must be as much authenticate as possible [15].

After suggesting EPAs by Ten Cate [4], these activities were described and their application was increasingly considered day after day; firstly in some professional medical specialties,and then, from 2013 to 2014 for general physicians in developed countries [16–19], and even other medical courses such as pharmacology [20]. It has increasingly become spread in medical education literature. EPAs are essential activities of any discipline (profession, specialty or sub specialty program), which the professionals must perform independently without direct supervision in a defined environment after acquiring appropriate competencies [3]. They are the units of the observable clinical works [21] and play a guiding role for clinical teachers for training and assessing the learners so as to make decision about the level of their entrustability and capability to accomplish the activities without close supervision. Ten Cate has stratified entrutability into five levels [6]. In the first level, the health professional learner is mainly an observer and is not allowed to get involved in the activity. The second level corresponds to the one in which (s) he may undertake the activity under close and active supervision by the clinician. In the third level, the learner performs the activity with indirect supervision and the clinician involves if needed. The learner performs the task independently without any supervision in the fourth entrustability level. And finally, in the fifth level, (s) he is eligible to supervise lower level learners. The EPAs were originally developed for graduate medical education (GME) [22–24]. Beeson at al. defined EPAs for emergency medicine [25]. Psychiatry, family medicine, anesthesiology and surgery were also the other programs for which the EPAs have been developed [26–29]. However, the gap perceived in continuum from undergraduate medical education (UGME) to GME, resulted in developing some EPAs for entering residency in addition to EPAs for undergraduate programs [16, 18, 30–33]. Moreover the sociocultural differences of the societies and health delivery systems in different countries and educational institutions have resulted in diversity of EPAs both in their numbers and contents. The United States has defined 13 EPAs for undergraduate medical education [34], while in Canada, despite using the American EPAs as the prototype, the number was decreased to 12 [35]. Besides to the number and content, there is another profound difference in terms of centrality and decentralization of the ways of developing EPAs. The USA and Canada centrally defined EPAs [34, 35] while Utrecht University in the Netherlands, Charit University in Germany and the Medical School of California in San Francisco developed their own EPAs [36–38]. As the result, EPAs are concepts that should be defined and customized for any community in order for training and assessing their health professionals, based on the needs. One of the main causes of the diversity in developing and implementing the EPAs among different communities might be stemmed from the differences in educational systems of undergraduate and graduate medical education. Such differences exist in admission, training programs, and even the rules and regulations of entering the graduates to practice. The present study, therefore, is going to determine what essential activities internal medicine residents have in developing countries like Iran. And at which entrustability level, they must be at the end of each residency year. The findings could be applied in all countries with similar situations, especially in East Mediterranean Regional Office (EMRO) countries.

### Setting

This study was conducted in Internal Medicine clinical department of Isfahan University of Medical Sciences, Isfahan, Iran, in order for developing the EPAs of internal medicine specialty residency program. After getting M.D. degree, the prospect residents take an annual national entrance exam. The participants are ranked regarding their scores and then-after they select their preferred specialty program. The admitted residents spend 4 years in a mostly hospital based graduate program. Besides formative evaluations throughout the program, they must take a progress test at the end of each year. Promoting to the higher level of study is based on their progress test scores. If they get the minimum pass level, required for the progression, they are eligible for moving to the next residency year, and if not, they are not allowed and must repeat that level. At the end of the fourth year, residents take another annual national final exam for getting the board certificate.

Internal medicine department of Isfahan University of Medical Sciences, school of medicine, with 61 clinical faculty members, trains 80 specialty residents each year within four residency years. The residents take various sub-specialty and general internal medicine rotations and the rotations take 1 month in length.

## Methods

Different studies followed different methodologies for developing EPAs. Ten cate et al. have suggested three steps for defining the EPAs as identification, processing, and validating [39, 40]. We extend them to six steps as follows:

1. Determining the characteristics of EPAs: These features were used as the main criteria for defining, processing and validating the EPAs in the following steps. Moreover, these features were applied for reaching to a shared language among the study team as well as with clinicians in order for accepting or rejecting any suggested statement.
2. Reviewing the literature and publications through a scoping review: This step was accomplished for fining the update EPAs developed specifically for internal medicine. We searched PUBMED, SCOPUS, and google scholar for English publications about similar experiences from 2005 to 2020. We used these keywords: “entrustable professional activities” “internal medicine” “internship“ ”residency” “undergraduate”, and “fellowship”. We also reviewed the guidelines such as Accreditation Council for Graduate Medical Education (ACGME) publications [41–43]. PUBMED search was performed as following: *(Filters applied: Abstract, Free full text, Full text, Journal Article, in the last 5 years, Humans, English, MEDLINE): (entrustable professional activities)*. For SCOPUS data base we used this formula: *(TITLE-ABS-KEY (“entrustable professional activities”*) *AND TITLE-ABS-KEY (internal AND medicine) AND TITLE-ABS-KEY (internship) OR TITLE-ABS-KEY (residency) OR TITLE-ABS-KEY (undergraduate) OR TITLE-ABS-KEY (fellowship*). In Google Scholar the following string were used*: entrustable professional activities“ AND (residents OR interns) And “internal Medicine”.* We included only those publications that were about the specialty program of general internal medicine but not sub-specialties or undergraduate level of internal medicine like clerkship. We excluded also those cases that reported a limited part of the curriculum like discharging the patient. The screening results were crossed over between the first and second authors.
3. Developing initial EPAs: With consideration of the national and institutional internal medicine residency curriculum [44, 45] as well as the residents’ electronic logbook (mobile log application), these documents were also reviewed for developing initial EPAs.
4. Extracting the results of preceding steps: We screened the activities, EPAs and other results, yielded from step 1st to 3rd, and developed the initial list of EPAs.
5. Refining and confirming the final EPAs: We formed a focus group discussion with a number of internal medicine clinicians. The criteria for participation were as: being internal medicine specialist with direct contribution in general internal medicine residency training, having at least 10 year experiences in teaching and assessment of internal medicine residents, being professor or associate professor, being interested in improvement of the residency program with conducting at least one educational research project or two papers in educational journals. The head of internal medicine department nominated 20 academic members who had our inclusion criteria. Since the optimal number of focus group participants is six to twelve [46], we invited 10 clinicians for the session. Informed verbal consent to participate was obtained from all participants at the beginning of the session. They participated in refining and screening the initial list as well as the entrustability level of each residency year. The focus group also determined the information sources, eligible for evaluating the residents against the EPAs. As the final section of the meeting, the focus group cross-tabbed the EPAs with competencies. For this purpose, we used the competencies, stablished by secretariat of council of general practitioners’ education for undergraduate medical education. They include clinical skills, communication skills, patient care (diagnosis, treatment and rehabilitation), health promotion and preventive medicine, and the G.Ps’ role (in that, personal development and continuous learning, professionalism, ethics and medical law, and finally, decision making, reasoning and problem solving skills are considered).
6. Reaching the final number and content of the EPAs: As the final step, we sent an open ended questionnaire attached to the EPAs to those nominated academics who did not attend the focus group session. We sent them via email and asked participants to evaluate and endorse the content validity of the focus group resulted EPAs based on the characteristics yielded in the first step. They were also asked to confirm the entrustability levels and EPA-competency matrix. Eventually, we reached the final number and content of the EPAs, based on the conclusions from all resources.

## Findings

**Fig. 1:**
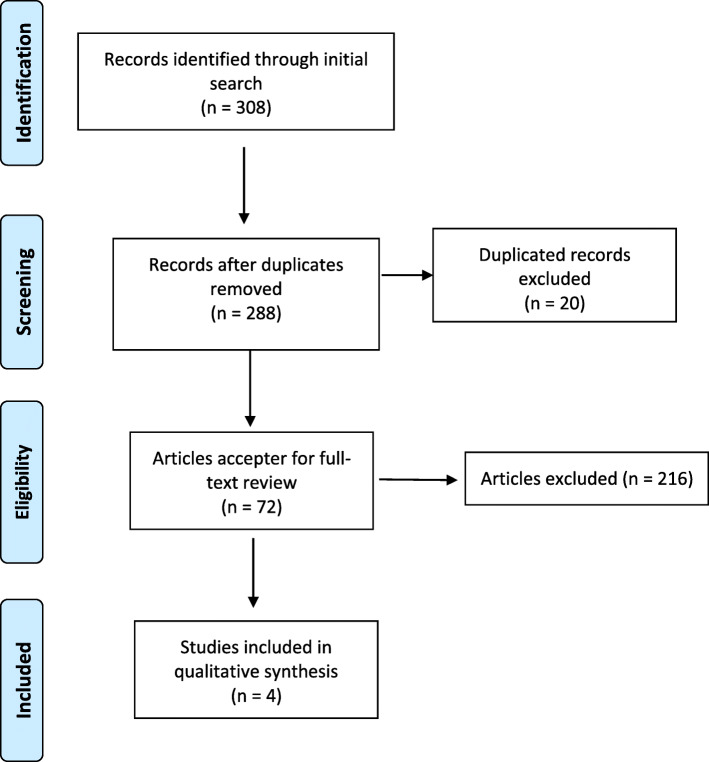
flow diagram of internal medicine EPAs scoping review

Within the first step, we determined the characteristics and features of EPAs including being parts of the profession, being directly related to the patient, and being observable, repetitive, assessable, doable by one resident, doable in a defined time frame, critical, and requiring especial knowledge, attitude and skills [3, 39].In the second step and through the scoping review, we found 308 items in the initial search; and eliminating duplicates resulted in 288 titles from which, after twice reviewing the titles and abstracts independently by the first and second authors, we selected 72 articles for a full text review. Those articles that were not in the internal medicine residency programs or those studying a limited area or a sub-specialty program were excluded. We also excluded the articles reporting the mere implementation of the EPAs. Figure 1 shows the quantity of the results in each step of search flow.

Only 4 articles studied developing EPAs in general internal medicine residency program [47–50]. Table 1 illustrates them in brief besides their resulted EPAs. The first one is the report of the initiative of the Education Redesign Committee of the Alliance for Academic Internal Medicine that reached to 16 EPAs. The next one was the result of a study in University of California in San Francisco (UCSF), and school of medicine. They devised a 30 item EPA table. The third study developed the EPAs for all 7 bed-side procedures of internal medicine residents. And the last but not the least, developed 29 EPAs throughout the four stages of internal medicine residency program (3 for entering stage, 7 for foundations, 11 for the core of the program, and finally, 8 titles for arriving at the practical work place).

**Table 1:** EPAs resulted in studies on internal medicine residency programs adapted from O’dawed et al. [51]

Author, year and Country of study	EPAs
Caverzagie et al., 201 5[47]. USA	1. Manage care of patients with acute common diseases across multiple care settings. 2. Manage care of patients with acute complex diseases across multiple care settings. 3. Manage care of patients with chronic diseases across multiple care settings. 4. Provide age-appropriate screening and preventative care. 5. Resuscitate, stabilize, and care for unstable or critically ill patients. 6. Provide perioperative assessment and care. 7. Provide general internal medicine consultation to nonmedical specialties. 8. Manage transitions of care. 9. Facilitate family meetings. 10. Lead and work within interprofessional health care teams. 11. Facilitate the learning of patients, families, and members of the interdisciplinary team. 12. Enhance patient safety. 13. Improve the quality of health care at both the individual and systems level. 14. Advocate for individual patients. 15. Demonstrate personal habits of lifelong learning. 16. Demonstrate professional behavior.
Hauer, Kohlwes, Cornett et al., 2013 [48] USA	1. Evaluate and manage a new problem in a continuity ambulatory patient requiring coordination of care between providers and across settings. 2. Admit and manage a medical inpatient with a new acute problem on a medical floor. 3. Admit and manage a medical inpatient with an acute exacerbation of a chronic problem on a medical floor. 4. Lead a family meeting to discuss serious or sensitive news with patient and/or family and other health providers. 5. Perform initial H&P, develop problem list, and plan for new ambulatory patient in continuity practice. 6. Provide continuity care, conducting interval visits, for primary care patients with multiple chronic conditions. 7. Develop and implement a safe discharge plan for a patient from the acute care setting. 8. Discuss serious news with a patient and/or family (bad news, end-of-life care planning). 9. Provide continuity care, conducting interval visits, for primary care patients. 10. Triage medically ill patients to an appropriate level of care. 11. Access medical information to provide evidence-based care for adult patients. 12. Identify and manage acute, emergent problems. 13. Provide urgent and emergent cross-coverage care to medicine inpatients. 14. Lead a team in managing multiple inpatients. 15. Recognize and diagnose common non-internal medicine (surgical, neurological, dermatologic, etc) problems and appropriately refer to subspecialty care. 16. Diagnose conditions for and co-manage patients with complex problems needing subspecialty care (inpatient or outpatient). 17. Manage information and knowledge for personal learning to improve care delivery and to educate others (journal club, etc.) 18. Institute palliative care appropriately in collaboration with palliative care specialists. 19. Perform behavioral counselling with a patient. 20. Provide medical consultation for patients receiving nonmedical services. 21. Admit and manage a medical ICU patient. 22. Identify and address a quality improvement need in a clinical setting. 23. Provide telephone management of an acute problem for an ambulatory patient. 24. Provide care to an inpatient or outpatient non-English speaking patient, using appropriate translator services. 25. Develop and implement an action plan based on review of performance data for one’s ambulatory patient panel. 26. Provide inpatient and outpatient care for patients with challenges in access to care that inappropriately address those challenges. 27. Conduct or participate in a scholarly project (research, QI, education, other). 28. Participate and believe an inpatient cardiopulmonary resuscitation. 29. Provide initial management and contribute to postoperative care for patients presenting with surgical problems. 30. Perform common procedures in internal medicine (LP, thoracentesis, central line, arthrocentesis).
Pugh et al., 2017 [49] Canada	1. Central venous catheter insertion. 2. Lumbar puncture. 3. Peripheral arterial catheter insertion. 4. Paracentesis. 5. Endotracheal intubation. 6. Thoracentesis. 7. Knee arthrocentesis.
Taylor et al., 2018 [50] Canada	Transition to Discipline 1. Performing histories and physical examinations and documenting and presenting findings across clinical settings for initial and subsequent care. 2. Identifying and assessing unstable patients, providing initial management, and obtaining help. 3. Performing the basic procedures of internal medicine Foundations of discipline 4. Assessing, diagnosing, and providing initial management for patients with common acute medical presentations in acute care settings. 5. Managing patients admitted to acute care settings with common medical problems and advancing their care plans. 6. Consulting specialists and other health professionals, synthesizing recommendations, and integrating these into the care plan. 7. Formulating, communicating, and implementing discharge plans for patients with common medical conditions from acute care settings. 8. Assessing unstable patients and providing targeted treatment and consulting as needed. 9. Discussing and establishing patients’ goals of care. 10. Identifying personal learning needs while caring for patients and addressing those needs. Core of Discipline 11. Assessing, diagnosing, and managing patients with complex or atypical acute medical presentations. 12. Assessing and managing patients with complex chronic conditions. 13. Providing internal medicine consultation to other clinical services. 14. Assessing, resuscitating and managing unstable and critically ill patients. 15. Performing the procedures of internal medicine. 16. Assessing capacity for medical decision making. 17. Discussing serious and/or complex aspects of care with patients, families, and caregivers. 18. Caring for patients who have experienced a patient safety incident (adverse event). 19. Caring for patients at the end of life. 20. Implementing health promotion strategies in patients with or at risk for disease. 21. Supervising junior learners in the clinical setting. Transition to practice 22. Managing an inpatient medical service. 23. Managing longitudinal aspects of care in a medical clinic. 24. Assessing and managing patients with uncertain diagnosis and/or treatment. 25. Providing consultations to off-site healthcare providers. 26. Initiating and facilitating transfers of care through the health care system. 27. Working with other physicians and healthcare professionals to develop collaborative patient care plans. 28. Identifying learning needs in clinical practice and addressing them with a personal learning plan. 29. Identifying and analyzing system level safety, quality, or resource stewardship concern in health care delivery.

In the third step, the national and institutional curricula were reviewed. The national curriculum has listed the internal medicine residents’ activities in their diverse roles including preventive, diagnostic-therapeutic, care giving, educational, research, and administrative roles. Moreover, these programs have included the cognitive and procedural skills for residents. In the institutional curriculum of Isfahan University of Medical Sciences, the activities, assigned to each residency year, were also indicated. And lastly, the log book, installed on the residents’ cellphones and mainly planed for procedural activities, was utilized as another source of initial EPAs.

At the end of the fourth stage, 41 activities were resulted as the initial EPAs for all four residency years. These titles had a profound variation in terms of both the content and nature. So, they needed to be more elaborated.

In the fifth stage, 10 clinical academics accepted to attend the meeting. Twenty-eight activities were developed as the EPAs for all four residency years. Table 2 shows the results. These EPAs cover all aspects of the internal medicine residency program. They consist of all roles and responsibilities of an internal medicine specialist including the therapeutic, educational, consultation, procedural and administrative roles.

**Table 2:** Internal medicine residency EPAs and their entrustability levels^a^ for each year

Num.	EPAs	Yr1	Yr2	Yr3	Yr4
1	Managing the internal medicine emergency cases (e.g. diabetic ketoacidosis, GI bleeding etc.)	2	3	4	5
2	Managing the cardiac emergency cases	2	3	4	5
3	Leadership of Internal medicine emergency wards especially at the times of crisis	2	3	4	5
4	Leadership of the health care (treatment) team	2	3	4	5
5	Cardio pulmonary resuscitation	3	4	5	5
6	Internal medicine history taking & physical exam	4	5	5	5
7	Clinical judgement & differential diagnosis	2	3	4	5
8	Interpreting the Para-clinical findings	2	3	4	5
9	Developing therapeutic protocols	2	3	4	5
10	Drug administration	2	3	4	5
11	Patient discharge	1	2	3	4
12	Admitting patient from other medical centers	1	2	3	4
13	Referring the patient to other services	1	2	3	4
14	Admitting patient from other services	1	2	3	4
15	Consultation to other services	1	2	3	4
16	Pre-operative consultation	1	2	3	4
17	Requesting consultation from other services	1	2	3	4
18	Appropriate Patient education	2	3	4	5
19	Teamwork collaboration with other services	2	3	4	5
20	Continuous competence improvement	2	3	4	5
21	Education to junior levels	2	3	4	5
22	Managing the common internal medicine outpatient cases	2	2	3	4
23	Visiting and care giving to patients in intensive care wards	2	3	4	5
24	Requesting appropriate diagnostic para-clinical investigations	3	4	5	5
25	Administering and requesting appropriate rehabilitations	2	3	4	5
26	Team leadership in the specialty field	2	3	4	5
27	Performing primary procedures (e.g. NGT insertion, urine smears preparation and examination etc.)	3	4	5	5
28	Performing advanced procedures (e.g. Lumbar Puncture, Pleural or ascites fluid tap etc.)	2	3	4	4

^a^Entrustability levels: 1: Be present and observe, 2: Act with direct supervision 3: Act with indirect supervision, 4: act without supervision 5: provide supervision

At the end of the focus group meeting, the EPAs were cross-tabbed with competencies. The related competencies to each EPA are shown in Table 3. Determining the appropriate evaluators was the final topic to be discussed in the meeting. The current and the last supervisors and chief residents all agreed up on as the key informants to evaluate residents against the EPAs using a common instrument.

**Table 3:** EPA-competency matrix

EPA Numbs	Clinical Skills	Communication Skills	Patient care	Health promotion and preventive medicine	Personal development and continuous learning	Professionalism, ethics and medical law	Decision making, reasoning and problem solving
1	*	*	*			*	*
2	*	*	*			*	*
3		*				*	*
4		*				*	*
5	*	*	*			*	*
6	*	*				*	
7	*				*		*
8					*	*	*
9	*	*	*		*	*	*
10	*	*	*	*	*	*	*
11		*		*		*	*
12		*			*	*	*
13	*	*	*	*	*	*	*
14	*	*	*			*	*
15	*	*	*		*	*	*
16		*			*	*	*
17	*	*	*		*	*	
18		*		*		*	
19	*	*	*		*	*	*
20	*				*	*	*
21		*	*	*	*	*	*
22	*	*	*	*	*	*	*
23	*	*	*		*	*	*
24	*	*	*	*	*	*	*
25	*	*	*	*	*	*	*
26	*	*	*	*	*	*	*
27	*	*	*		*	*	
28	*	*	*		*	*	

In the last step of the study, for content validity of the results, after follow up, all of the ten academic internists responded to our email. Their recommendations and suggestions were discussed in the research team meeting, and eventually, EPAs, entrustability levels, and EPA-competency matrix were finalized.

## Discussion

In this study we developed and finalized 28 EPAs for internal medicine residents. We also determined the level of entrustment at the end of each residency year and the interrelation between the final EPAs and those competencies expected from medical doctors in Iran. We already knew that different studies developed EPAs in different ways [6]. However, collecting, refining and validating are more common steps in most of the publications [51]. Gathering the initial statements, like the ones in our study, has been based on the literature review and group discussions, followed by revising and filtering steps. In this way, Hauer et al. only collected the opinions of the chief residents for processing the initial titles [38]. Caverzagie et al. mainly investigated the experts’ ideas [47], while in other studies, the ideas of learners in different levels have been scrutinized. For example, in Australia and New Zealand, the first and second year psychiatric residents’ EPAs were developed by using the fellowship residents’ suggestions [26]. However, in other programs like pediatrics, radiology, and anesthesia, clinical faculty members and the members of the central committees of the programs were invited [28, 52, 53].

The steps, followed in this study, were to some extent similar to the five steps, proposed by Kwan et al. including topic selection, content development, developing initial titles, getting feedback from stakeholders, and filtering and finalizing the activities [54]. Though, different ways such as comparing to other programs and conducting Delphi rounds have been suggested for collecting the initial titles [48].

Our final list of 28 EPAs is in accordance with the range of 20 to 40 titles, suggested by Ten Cate [6]. Although some authors reported much fewer EPAs like Chan et al. that studied only the patient handover [55], other studies developed more activities [56]. Moreover, there are some other kinds of study that classified the EPAs. For instance, Fessler et al. used three groups as pulmonary cares, pulmonary critical cares, and combination of these two groups of activities [57]. Graafland et al. developed 66 EPAs for sub divisions of anesthesiology as anesthesiology, emergency, gastroenterology, general surgery, gynecology, psychiatry and radiology [58].

Considering the EPAs listed in Table 2, it is inferred that these EPAs are not similar with regard to the time of entrustment. For example, patient admission and discharge is an activity which is done independently and without supervision only in the fourth year of residency; while, history taking and physical examination are the ones on which a second year resident must be capable to supervise the junior learners. Besides to these, the issue of what is professional activity, what is a task, what is a competency, and what is a competency category is difficult to parse -- even ten Cate seems to waiver in his descriptions of specificity in his various papers. Somewhere between sweeping general statements that cannot be directly measured (“the resident will have good patient care skills”) and specific, measurable tasks (“the resident will be able to perform a paracentesis”) lie the professional activities are unique to physicians in that specialty, are observable and measurable, and are part of the essential professional work of the specialty and not a general medical ability [7].

One of the advantages of our study is nominating the evaluators, which is noticeable with regard to the scarcity of the implementation and evaluation studies on EPAs. Nonetheless, peter et al. working on this phase of EPA study suggested using available information resources for assessing the residents [59].

Another advantage of the current study is determining the entrustability level of different residency years. It is while Mink et al. merely defined the meaning of each entrustability level in pediatrics fellowship program [60]. Hauer et al. only explored the perceptions of the faculty members and residents about the year a resident becomes entrustable on a given EPA [48].

There is another advantage for this study which is the comprehensiveness for all aspects of the residency program including the settings and competency domains; whereas, many studies reported development of EPAs in very limited areas of internal medicine graduate study like safe patient discharge [49, 61].

As shown in the competency-EPA matrix (Table 3), most of the EPAs are such broad that are related to many competencies. It means that each EPA is necessary for meeting more than one competency. It would absolutely be indicative of the inclusiveness of each EPA. It is in accordance with the Ten Cate’s suggestion [6]. He has mentioned elsewhere that the breadth of EPAs depends on the time they are used. For summative end of course decision making purposes named as “statements of awarded responsibility (STARS)” we need broader EPAs than formative uses throughout the course. For the latter we need to define nested EPAs [39]. As a result, and since we aimed to devise an end of year EPA list, some of them are more inclusive. It is suggested as next the step to implement the resulted EPAs in a pilot study so that they could be incorporated to a broader extent such as the national exams. Moreover, our further work would be defining the nested EPAs for broad ones.

## Limitation

One of the limitations of this study could be using no residents’ points of view; nonetheless, we considered the residency logbook contents.

Another limitation of our study is being a single institutional initiative. Using the viewpoints of academics and residents of other medical universities is also suggested.

## Data Availability

The datasets used and/or analysed during the current study available from the corresponding author on reasonable request.
